# Identification of genes differentially expressed between prostrate shoots and erect shoots in the lycophyte *Selaginella nipponica* using an RNA-seq approach

**DOI:** 10.1093/aobpla/plac018

**Published:** 2022-05-05

**Authors:** Jun Sun, Gui-Sheng Li

**Affiliations:** Laboratory of Plant Resource Conservation and Utilization, Jishou University, Jishou 416000, China; Laboratory of Plant Resource Conservation and Utilization, Jishou University, Jishou 416000, China

**Keywords:** Reproductive growth, *Selaginella nipponica*, *SPL* genes, spore development, transcriptome

## Abstract

Lycophytes are the earliest vascular plants and *Selaginella* is the most studied genus among them. Prostrate shoots are produced during early growth and erect shoots emerge later in *S*. *nipponica*, thus providing an opportunity for exploring the evolution of the mechanism underlying the transition between growth phases. Six libraries were sequenced for the prostrate and the erect shoots, and a total of 206 768 genes were identified. Some genes were differentially expressed in prostate and erect shoot, with relatively high expression in the prostate shoots being related to hormone responses and defence reactions, while higher expression in the erect shoots was related to spore formation and shoot development. Some *SPL* genes possessed a miR156 binding site and were highly expressed in the erect shoots, while *AP2*-like genes were more highly expressed in the prostrate shoots but simultaneously lacked any miR172 binding site. MiR156 was detected at a higher concentration in the prostrate shoots. Thus, the mechanism for the vegetative to reproductive transition of sporophytes probably originated in the common ancestor of vascular plants and must have experienced stepwise development during evolution.

## Teaser

The lycophyte *Selaginella nipponica*produces more transcripts of some *APETALA2*-like genes in the vegetative prostrate shoots than in the reproductive erect shoots, while this situation is inverse for some *SQUAMOSA PROMOTER-BINDING PROTEIN-LIKE*genes. The latter are expressed anti-parallel with miR156 during this growth transition, while there is no miR172 or its binding sites on putative target genes as in flowering plants. Thus, the genetic control of the vegetative to reproductive transition must have progressively developed during evolution.

## Introduction

Flowering plants generally experience two transitions of growth phase, in which they first shift from juvenile to adult with alterations in leaf size and shape, node length and trichome distribution (Huijser and Schmid 2011). The second transition is from the vegetative to reproductive stage, with the shoot apical meristem (SAM) ceasing stem growth and beginning to produce flowers. The *SQUAMOSA PROMOTER-BINDING PROTEIN-LIKE* genes (*SPL*s) encode proteins that can bind the promoter of the *SQUAMOSA* gene via their 76-aa SBP domains in *Antirrhinum majus* (Klein *et al.* 1996). There are 17 *SPL*s in *Arabidopsis thaliana*, and among them 11 have a miR156 binding site that can mediate transcript slicing and/or translation repression (Axtell and Bartel 2005). *SPL3*/*4*/*5* have a miR156 binding site located in the 3ʹ untranslated region (UTR), and when they are overexpressed as versions resistant to miR156 binding in *A. thaliana* they show early flowering (Cardon *et al.* 1997; Wu and Poethig 2006). However, overexpression of the wild-type *SPL3* leads to even earlier flowering, revealing that miR156 is a powerful inhibitor (Wu and Poethig 2006). Double mutation of *SPL9*/*15* can repress the transition from juvenile to adult in *A*. *thaliana*, while enhancing *SPL9* expression will eliminate the juvenile phase (Wang *et al.* 2009; Wu *et al.* 2009). Although *SPL2*/*10*/*11* function in the transition within embryo development, they also play a minor role in promoting transition from vegetative to reproductive growth in *A*. *thaliana* (Nodine and Bartel 2010). While *SPL13* also functions in transition from juvenile to flowering stage, *SPL6* is involved in other activities (Xu *et al.* 2016). *SQUAMOSA PROMOTER-BINDING PROTEIN-LIKE* genes are highly expressed upon flowering in *A. thaliana* (Schmid *et al.* 2003). In contrast, miR156 expression peaks in seedlings (Xu *et al.* 2016).

There are five *MIR172* loci in *A*. *thaliana*, and they are weakly expressed in vegetative growth and strongly expressed in reproductive growth; thus, the level of proteins and/or transcripts of their targets, the *APETALA2* (*AP2*)-like genes, fluctuate inversely during growth (Aukerman and Sakai 2003; Chen 2004; Zhu and Helliwell 2011). It is interesting that *MIR172D* is highly expressed in SAMs while *MIR172A*/*B* is only expressed in leaf vasculature (Lian *et al.* 2021; O’Maoileidigh *et al.* 2021). *MIR172* overexpression causes early flowering (Aukerman and Sakai 2003; Chen 2004), while overexpression of *AP2*-like targets of miR172 represses flowering (Aukerman and Sakai 2003; Lauter *et al.* 2005). Moreover, loss of function of *AP2*-like genes accelerates flowering Evans *et al.* 1994; Moose and Sisco 1994; (Aukerman and Sakai 2003). Finally, *MIR172* genes can be activated by *SPL*s (Wu *et al.* 2009), and accordingly the miR172 level is greatly reduced in the *spl15* mutant (Hyun *et al.* 2016).

**Figure 1.**
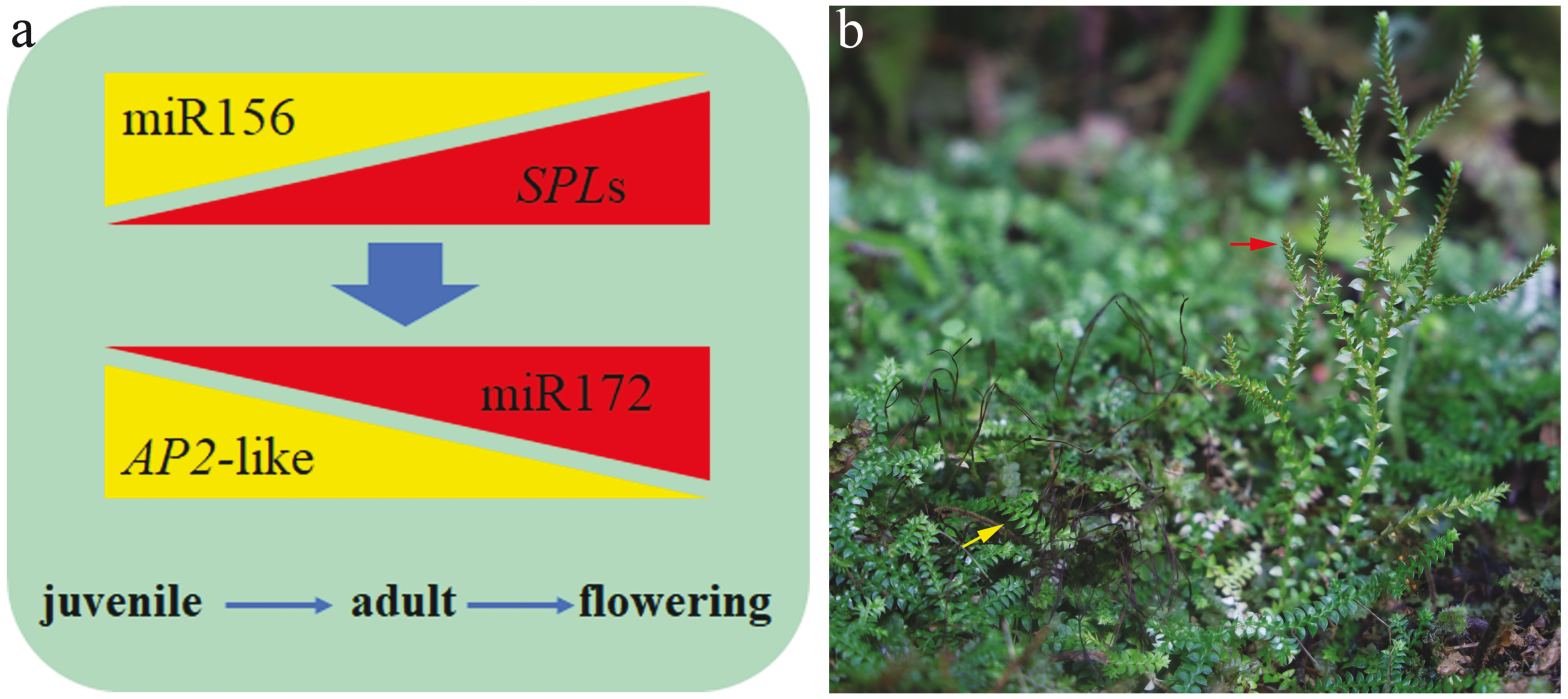
Genetics of growth phase transition in *Selaginella nipponica*. (A) Genes regulating phase transition in flowering plants. Regulation of genes by miRNAs is depicted by pairs of triangles pointing in opposite directions, and activation of *MIR172* by *SPL*s is shown by a wide arrow; the three main growth phases of flowering plants are presented below. (B) *Selaginella nipponica* growing in natural habitat. Prostrate shoots are indicated by the yellow arrow and erect shoots by the red arrow.

Lycophytes are sporophyte-dominant like flowering plants, but the former diverged 400 million years ago (MYA) (Banks *et al.* 2011). *Selaginella* is a lycophyte and produces sporangia in fertile shoots (Bonacorsi and Leslie 2019). There are two types of shoots in *Selaginella nipponica*, namely early prostrate shoots and later erect shoots, which can generate yellowish megaspores and reddish microspores (Fig. 1). Moreover, there are abundant rhizophores in the prostrate shoots while microphylls are sparsely arranged in erect shoots. Thus, this species provides an opportunity for the evolutionary study of developmental genetics, and particularly for exploring the conservation of miR156–SPL and miR172–AP2 interactions in vascular plants (Fig. 1). This study sequenced and analysed the transcriptomes of prostrate and erect shoots in *S*. *nipponica* and found that genes may be differentially expressed in the prostrate and erect shoots, which suggested some distinctive activities occurring in each type of shoot. *SQUAMOSA PROMOTER-BINDING PROTEIN-LIKE* genes were weakly expressed in the prostrate shoots but highly expressed in the erect shoots. In contrast, *AP2*-like genes were more highly expressed in the prostrate relative to the erect shoots.

## Materials and Methods

### Collection of specimens

*Selaginella nipponica* specimens were collected from a single population growing in a natural habitat at 6:00 pm (Beijing Time Zone) in May 2019, with the prostrate shoots and the erect shoots including sporangia being separately harvested into liquid nitrogen. Samples were then stored at −80 °C and transported on dry ice. Three subsamples of each sample were taken, which served as the biological replicates.

### Sequencing of transcriptomes

A CTAB method was employed to extract RNA for transcriptomic analysis (Jordon-Thaden *et al.* 2015), and the Hiseq X platform was used for sequencing (Illumina, San Diego, CA, USA). Libraries were constructed via mRNA purification and fragmentation, cDNA synthesis with random primers, and adaptor ligation and filtration. Sequencing was performed on both ends, producing two reads around 150-bp long for every clone insert. The Trinity v2.0.6 software ‘min_kmer_cov 2’ was used to assemble transcripts (Haas *et al.* 2013), and the longest transcripts were designated as unigenes. NR (http://ncbi.nlm.nih.gov/), SwissProt (http://www.gpmaw.com/html/swiss-prot.html) and TrEMBL (https://www.uniprot.org/news/2004/03/02/full) databases were used to identify conserved proteins, while TransDecoder was used to annotate novel unigenes. BUSCOv5.1.3 was run under the transcriptome mode and against viridiplantae_odb10 (Simao *et al.* 2015). Clean reads were stored in the National Center for Biotechnology Information (NCBI, Bethesda, MD, USA) with the accession number GSE164112.

### Identification and annotation of differentially expressed genes

Read counts of unigenes were supplied to DESeq2 (http://www.r-project.org/). Differentially expressed genes (DEGs) between the prostrate shoots and the erect shoots were determined according to the adjusted *P*-value (namely *Q*-value) < 0.05 and |log2FoldChange| > 1. Preferentially expressed genes (PEGs) for a certain sample were DEGs that were upregulated in that sample. Functional annotation of PEGs was carried out using Uniprot (https://www.uniprot.org), KEGG (http://www.kegg.jp) and COG (https://www.ncbi.nlm.nih.gov/COG/). Expression level was calculated as transcripts per million reads (TPM) for each unigene.

### Construction of phylogenetic trees

The blastp program was used and an empirical *E*-value of 1*e*-005 was used to collect similar sequences under multiple queries including *SPL3* from *A*. *thaliana*. The hmmer3.0 program was used to identify SBP domains and CLUSTAL W was used to align them (Thompson *et al.* 1994). The FastTree program was used to construct phylogenetic trees (Price *et al.* 2009). To search for miR156 and miR172 binding sites, transcript sequences of genes were retrieved from TAIR (https://www.arabidopsis.org), Ensembl Plants (http://plants.ensembl.org/index.html), FernBase (https://www.fernbase.org) and FIMO (http://meme-suite.org/tools/fimo) (‘fimo --oc --verbosity 1 --thresh 1.0E-4’).

### Reverse transcription quantitative polymerase chain reaction

cDNA was synthesized from RNA remaining from the construction of transcriptome libraries, with the aid of the PrimeScript II reverse transcriptase (TAKARA, Beijing, China) and the poly(T) primer. The TB Green Advantage qPCR Premix (TAKARA) was used, and a 10 μL reaction system was constituted by 1.8 μL water, 5 μL Premix, 0.2 μL ROX dye, 2 μL pairs of primers and 1 μL template cDNA. The PCR procedure consisted of an initial denaturation at 95 °C for 7 min, followed by 45 cycles at 95 °C for 5 s, 60 °C for 30 s and a final dissociation. The instrument used was an PikoReal fluorescence reverse transcription quantitative polymerase chain reaction (RT-qPCR; Thermal Fisher Scientific, Shanghai, China). The threshold Cq (Ct) was subtracted for tested genes by referring to an actin gene, and relative expression levels were calculated using the 2^−ΔCt^ method, thus providing data for statistical analysis and fold-change computation (Schmittgen and Livak 2008). Prism (GraphPad Software, San Diego, CA, USA) was used for unpaired Student’s *t*-tests and to calculate the one-tailed *P*-value. For miR156, small RNA fragments were prepared from fresh total RNA using RNAzol (MRC, Cincinnati, OH, USA), and mature miRNA was added to a poly(A) tail and then reverse-transcribed using mRQ Enzyme Mix (TAKARA). A U6 gene was employed to control the expression level. RT-qPCR used the same three biological replicates from the prostrate and erect shoots, and there were at least three technical replicates for each of them.

## Results

### Transcriptomes of the prostrate and erect shoots

**Figure 2.**
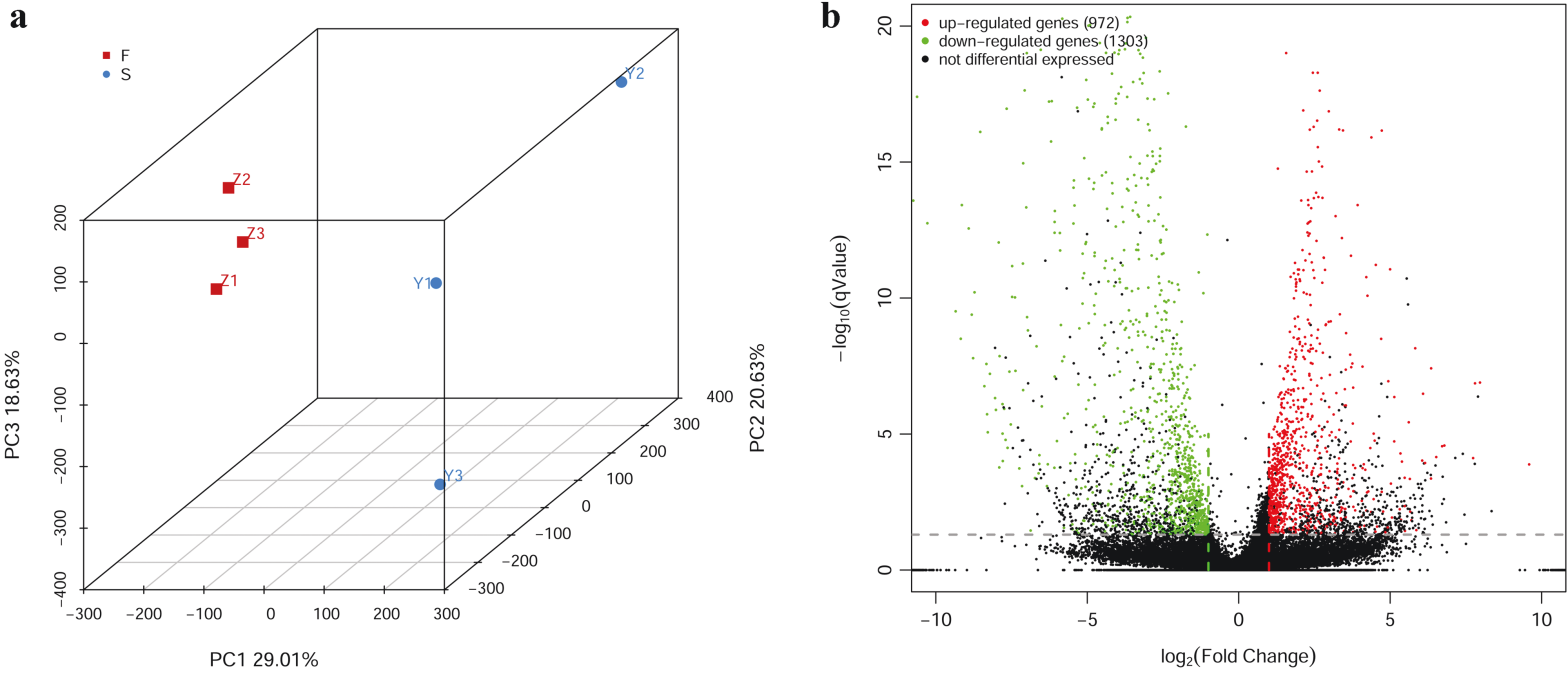
Analysis of TPM values and counts. (A) Principal component analysis of TPM values. Names of samples are shown. (B) Comparison of unigenes from the prostrate and the erect shoots based on read counts.

Three libraries each were sequenced for the prostrate shoots and the erect shoots of the lycophyte *S*. *nipponica*, and the results were combined to provide a transcriptome for each type of shoot (Table 1). A total of 206 768 unigenes were identified, and 91.6 % of the clean reads could aligned to these unigenes. Of these, 94.3 % unigenes were assigned an ortholog in the BUSCO analysis, with 80.9 % unigenes being assigned a complete and single copy ortholog, 0.9 % a complete and duplicated ortholog and 12.5 % a fragmented ortholog. Unigenes were long as 469 bp in terms of N50 and 222 bp in N90, with a maximum of 25 386 bp, minimum of 201 bp and average of 438 bp. There were 185 363 unigenes that potentially code for proteins, 107 452 of which might encode conserved proteins. The expression level was constant for unigenes across biological replicates but differed between samples (Fig. 2A). There were numerous DEGs (Fig. 2B), including 972 unigenes that were upregulated in the prostrate shoots and thus considered PEGs. Likewise, 1303 unigenes were upregulated in the erect shoots and were their PEGs.

**Table 1.** Basic data of the transcriptomic sequencings. Y1, Y2 and Y3 were three samples of the prostrate shoots, and Z1, Z2 and Z3 were three samples of the erect shoots.

	The prostrate shoots			The erect shoots		
	Y1	Y2	Y3	Z1	Z2	Z3
Raw reads	39 869 072	45 816 802	40 565 992	51 674 106	44 970 426	44 193 362
Clean reads	38 609 838	43 799 192	39 314 834	49 441 246	43 431 710	42 717 476
Read length	142.5	143.96	146.67	144.34	145.34	143.86
Q30 proportion	95.72 %	95.11 %	95.40 %	95.39 %	95.62 %	95.54 %
Ambiguity percent	0.00 %	0.00 %	0.00 %	0.00 %	0.00 %	0.00 %
GC content	51.06 %	51.01 %	50.90 %	51.48 %	51.18 %	51.00 %

### GO annotations of the PEGs

For PEGs in the prostrate shoots, GO:0009755 (hormone-mediated signalling pathway) had the highest significance among terms relating to biological process **[see****Supporting Information—Fig. S1A]**. Similar terms such as GO:0009725 (response to hormone), GO:0032870 (cellular response to hormone stimulus), GO:0071365 (cellular response to auxin stimulus) and GO:0009733 (response to auxin) also had a *Q*-value lower than 1.00*e*-07. GO:0009699 (phenylpropanoid biosynthetic process) and GO:0009698 (phenylpropanoid metabolic process) were also enriched to the same order of magnitude. GO:0006952 (defence response), GO:0031408 (oxylipin biosynthetic process) and GO:0031407 (oxylipin metabolic process) were the next most significant terms with a *Q*-value around 1.00*e*-06, but did not have a significant *Q*-value in the erect shoots. Terms relating to cellular components that ranked highest in the prostrate shoots included GO:0031224 (intrinsic component of membrane), GO:0016021 (integral component of membrane), GO:0044425 (membrane part) and several other terms concerning plastids and chloroplasts. Among these, GO:0046658 (anchored component of plasma membrane) had a larger *Q*-value in the prostrate shoots relative to the erect shoots (1.48*e*-06 vs. 1.53*e*-10). Among terms related to molecular function, GO:0046906 (tetrapyrrole binding) and GO:0020037 (haem binding) showed the most significant enrichment. GO:0003700 (DNA-binding transcription factor activity) and GO:0004497 (monooxygenase activity) showed high enrichment in the prostrate shoots but had *Q*-values close to or even higher in the erect shoots. GO:0042973 (glucan endo-1,3-beta-D-glucosidase activity) and several related terms showed significant enrichment in the prostrate shoots, whereas GO:0008422 (beta-glucosidase activity) had no *Q*-value in the erect shoots.

For PEGs in the erect shoots, the most significant term within the biological process category was GO:0048229 (gametophyte development), and the similar GO:0009555 (pollen development) was also highly enriched **[see****Supporting Information—Fig. S1B****]**. These were also evident in the prostrate shoots but with a much larger *Q*-value. For example, the *Q*-value for GO:0009555 was 1.10*e*-07 in the prostrate shoots and 1.90*e*-13 in the erect shoots. GO:0048608 (reproductive structure development), GO:0061458 (reproductive system development), GO:0010154 (fruit development) and GO:0009908 (flower development) were also significantly enriched in the erect shoots. GO:0048367 (shoot system development) had a *Q*-value of 1.76*e*-09 in the erect shoots and 0.0008 in the prostrate shoots, and the situation was the same for GO:0046373 (L-arabinose metabolic process). Among terms related to cellular components, GO:0005618 (cell wall), GO:0030312 (external encapsulating structure), GO:0012511 (monolayer-surrounded lipid storage body) and GO:0042735 (protein body) ranked highest in the erect shoots while they were absent in the prostrate shoots. Among terms related to molecular function, GO:0045735 (nutrient reservoir activity) and GO:0046556 (alpha-L-arabinofuranosidase activity) were significantly enriched in the erect shoots while they did not appear in the prostrate shoots.

### KEGG and KOG annotations of the PEGs

Preferentially expressed genes in the prostrate shoots were associated with four metabolic pathways: ko00940 (phenylpropanoid biosynthesis), ko04075 (plant hormone signal transduction), ko00592 (alpha-linolenic acid metabolism) and ko04016 (MAPK signalling pathway) **[see****Supporting Information—Fig. S2A****]**. Preferentially expressed genes in the erect shoots were associated with three metabolic pathways: ko00010 (glycolysis/gluconeogenesis), ko00040 (pentose and glucuronate interconversions) and ko00940 **[see****Supporting Information—Fig. S2B****]**. Preferentially expressed genes in the prostrate shoots were associated with four clusters of homologous proteins, namely Q (secondary metabolite biosynthesis, transport and catabolism), I (lipid transport and metabolism), E (amino acid transport and metabolism) and G (carbohydrate transport and metabolism). Preferentially expressed genes in the erect shoots were associated with six clusters of homologous proteins, namely Q, P (inorganic ion transport and metabolism), E, G, M (cell wall/membrane/envelope biogenesis) and L (replication, recombination and repair).

### Phylogeny and expression of *SPL*s and *AP2*-like genes

**Figure 3.**
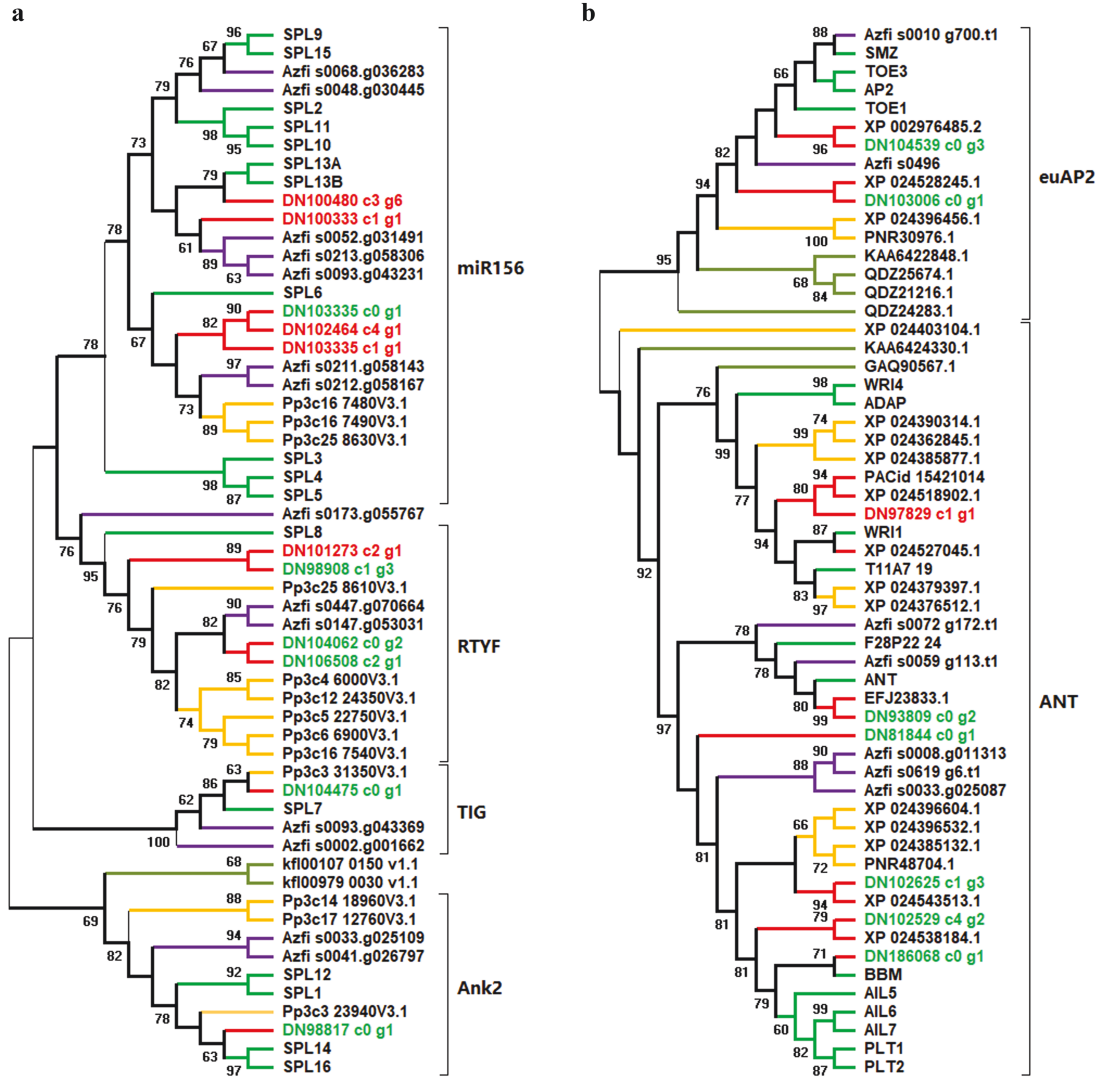
Phylogeny of *SPL*s (A) and *AP2*-like genes (B). Numbers along branches indicate support values for genes from *Arabidopsis* (green), *Azolla imbricate* (purple), *Selaginella nipponica* (red), *Physcomitrella patens* (yellow) and *Klebsormidium flaccidum* (olive). Genes from *S*. *nipponica* that were upregulated in the erect shoots are indicated in red, while those in the prostrate shoots are in green. Major clades are delimited by brackets.

*SQUAMOSA PROMOTER-BINDING PROTEIN-LIKE* genes are found in algae and constitute a gene family consisting of four major clades (Fig. 3A). The miR156 clade is the largest and characterized by the miR156 binding site. Genes within the RTYF clade possess a 13-aa motif before the SBP domain, as exemplified by RIGLNLGGRTYFS in SPL8 from *A. thaliana*. The Azfi_s0173.g055767 gene of the fern *Azolla imbircata* could not be unambiguously assigned despite possessing a miR156 binding site and simultaneously lacking the RTYF motif. The TIG clade contains proteins possessing a TIG-domain-like region before the SBP domain; in contrast, an Ank2-domain-like region follows the SBP domain in the Ank2 clade. There are two *SPL*s in the alga *Klebsormidium flaccidum*, among which kfl00107_0150_v1.1 lacks any of the above-mentioned conserved regions including the miR156 binding site, with the exception of the SBP domain, while kfl00979_0030_v1.1 resembles members of the TIG clade. There were 11 *SPL*s in our transcriptomes of *S*. *nipponica*, while just nine *SPL*s were found in the genome of *S*. *moellendorfii*, with DN100333_c1_g1 and DN102464_c4_g1 having no equivalent in the latter. Of these 11 *SPL*s, five were in the miR156 clade, four in the RTYF clade, one in the TIG clade and the last one in the Ank2 clade. However, DN100480_c3_g6 located within the miR156 clade did not possess a miR156 binding site in its complete coding sequence or in its 600-bp-long 3ʹ UTR. *AP2*-like genes of green plants including algae can be divided into the euAP2 and the ANT clade (Fig. 3B). There were eight *AP2*-like genes in *S*. *nipponica* while only seven such genes were found in the genome of *S*. *moellendorfii*, and there were two genes in the euAP2 clade in *S*. *nipponica*.

**Figure 4.**
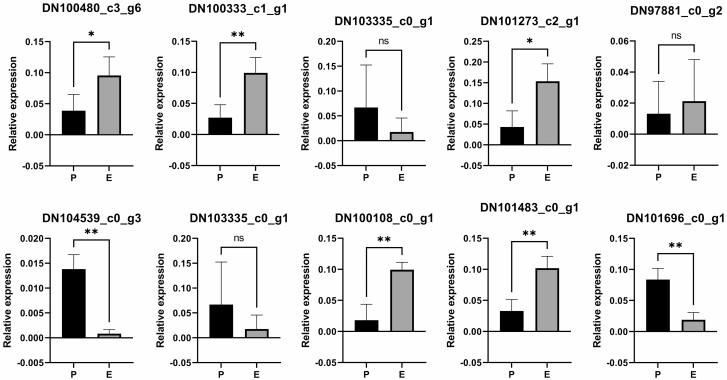
RT-qPCR validation. Genes were tested in the prostrate shoots (P) and in the erect shoots (E); relative expression and statistical symbols are shown, with *P < 0.05, **P < 0.01, ^ns^*P* > 0.01 in three biological replicates.

Among the *SPL*s, DN100480_c3_g6 had a mean TPM of 30.94 in the prostrate shoots and 46.22 in the erect shoots, but this difference was not significant. The situation was the same for DN100333_c1_g1. However, RT-qPCR revealed DN100480_c3_g6 was expressed about 3-fold higher in the erect shoots than in the prostrate shoots, and the situation was 7-folds for DN100333_c1_g1 (Fig. 4). DN103335_c1_g1 had a greater mean TPM value in the erect shoots albeit with an insignificant difference, and DN105179_c0_g2, which overlapped in sequence with DN103335_c1_g1, had a significantly larger TPM value in the erect shoots (*Q* = 3.87*e*-08). DN102464_c4_g1 had a mean TPM that was slightly higher in the erect shoots than in the prostrate shoots. Finally, DN103335_c0_g1 had a mean TPM of 5.64 in the prostrate shoots while this value was just 0.27 in the erect shoots; this difference was replicated in RT-qPCR. DN101273_c2_g1, which was outside the miR156 clade, was significantly preferentially expressed in the erect shoots in both RNA-seq and RT-qPCR. Meanwhile, miR156 was highly expressed in the prostrate shoots, with a fold change of about 16.95 (Fig. 4). For *euAP2* genes, DN104539_c0_g3 and DN103006_c0_g1 expression was 2-fold higher in the prostrate shoots relative to the erect shoots, and this difference was significant. The same pattern was observed for the former gene in RT-qPCR (Fig. 4). All *ANT* genes except for DN97829_c1_g1 were also preferentially expressed in the prostrate shoots according to the TPM value. Finally, DN97881_c0_g2 (which was close to *AGL67*), DN100108_c0_g1 (close to *CUC2*), DN101483_c0_g1 (close to *ARF2*) and DN101696_c0_g1 (close to a pathogenesis-related thaumatin gene) were differentially expressed between the two types of shoots in RNA-seq and/or RT-qPCR. Differences in DN97881_c0_g2 and DN103335_c0_g1 were not significant in terms of *P* < 0.05, possibly because the fold change was 1.07 and 2.47 for these two, respectively, whereas other genes had fold changes ranging from 2.47 in DN100480_c3_g6 to 16.94 in DN104539_c0_g3. A low peak of expression level might also be responsible for the weak statistical power in the comparison between DN103335_c0_g1 and DN100480_c3_g6.

## Discussion

Lycophytes were the first vascular plants and diverged from euphyllophytes (ferns and seed plants) 400 MYA (Banks *et al.* 2011), and thus they are important in understanding plant evolution. *Selaginella nipponica* is characterized by prostrate shoots and erect shoots, with the former being juvenile and the latter being capable of producing sporangia. It is therefore attractive for studying the evolution of the growth phase transition.

Transcriptomic analysis revealed that there are numerous DEGs between the two types of shoots in *S*. *nipponica*, and that PEGs of certain types of shoots hint at some biological aspects. For example, a hormone response was indicated by PEGs in the prostrate shoots. Meanwhile, DN99249_c0_g1 is homologous to genes affecting shoot and root development in *A*. *thaliana* (Nakazawa *et al.* 2001; Takase *et al.* 2003), DN93544_c0_g1 is homologous to genes responding to salt stress (Balsemao-Pires *et al.* 2011), DN105307_c0_g2 is homologous to genes involved in removing chemical contaminants from environments (Gunning *et al.* 2014) and DN105334_c0_g2 is homologous to genes involved in the uptake of mineral ions from soils (Jakoby *et al.* 2004; Yuan *et al.* 2005). Thus, hormones possibly have further affected development and adaption of the prostrate shoots. Nevertheless, roots branch through the bifurcation of meristems in *Selaginella* species, suggesting the lack of auxin-driven formation of the lateral roots (Matsunaga *et al.* 2017; Fang *et al.* 2019; Ferrari *et al.* 2020). The identified functions of phenylpropanoid and oxylipin probably provide resistance against fungus infection and worm nibbling in *S*. *nipponica* (Walter 1989; Johnson *et al.* 2019), which is consistent with the simultaneous identification of a defence response from PEGs in the prostrate shoots. KEGG analysis further suggested the prostrate shoots may use alpha-linolenic acid metabolism to cope with diseases (R. R. Singh *et al.* 2019; U. B. Singh *et al.* 2019), along with MAPK signalling to integrate various signals including hormones to co-ordinate development and defence (Zhang *et al.* 2018). Overall, these predictions are consistent with the morphological characteristics and habitat of the prostrate shoots of *S*. *nipponica*.

The most significant function of erect shoots is gametophyte and pollen development. Consistent with this, we found many PEGs homologous to genes responsible for pollen formation in flowering plants, such as *XRI*, which protects chromosomes from extensive fragmentation in meiocytes (Dean *et al.* 2009), and *ACOS5*, *CYP703A2* and *MS2*, which are required for sporopollenin monomer biosynthesis (Morant *et al.* 2007; de Azevedo Souza *et al.* 2009). *AGL67* is required for seed desiccation tolerance in *A. thaliana*, but it may regulate vegetative desiccation tolerance in early land plants (Gonzalez-Morales *et al.* 2016). Moreover, *SPL8* affects both microsporogenesis and megasporogenesis in *A. thaliana* (Unte *et al.* 2003). L-arabinose metabolism is unique to the erect shoots, suggesting it may promote spore production and inhibit their germination (Yan *et al.* 2020). Genes related to the formation of the cell wall, lipid and protein bodies and the nutrient reservoir were significantly enriched in the erect shoots, which also appears to be involved in sporogenesis. KOGs in the erect shoots are involved in the biogenesis of the cell wall/membrane/envelope and replication/recombination/repair activity, which is apparently critical to mitosis and miosis during spore formation. Shoot development function was also significant in the erect shoots, and the involved homologs included *Arabidopsis MYB33*, which is essential for normal shoot formation but can retard root development (Millar and Gubler 2005; Xue *et al.* 2017), and *Arabidopsis ZP1*, which can inhibit root hair initiation and elongation (Han *et al.* 2019). Consistently, fewer rhizophores are found in the erect shoots relative to the prostrate shoots. Finally, the *CUC2* gene forms part of a pathway controlling the number of the lateral branches in *A. thaliana*, and *CCD8* is critical to strigolactone biosynthesis, which can repress branching (Umehara *et al.* 2008). This is interesting since the prostrate shoots of *S. nipponica* seem to be indeterminant while the erect shoots are determinant.

*SQUAMOSA PROMOTER-BINDING PROTEIN-LIKE* genes can be divided into four major clades characteristic of different domains in land plants, while there are two *SPL*s in the alga *K*. *flaccidum* with one containing a TIG domain and the other lacking any characteristic motifs other than the SBP domain. It is possible that the common ancestor of all green plants including algae contained two *SPL*s, with one leading to the TIG/Ank2 clade and the other to the RTYF/miR156 clade (Guo *et al.* 2008; Li *et al.* 2020). In the miR156 clade, there is one gene cluster in the moss *Physcomitrella patens*, two clusters in the lycophyte *S*. *nipponica*, three clusters in the fern *A*. *imbricate* and four to five clusters in the flowering plant *A*. *thaliana*, revealing that clusters in this clade have increased in number during evolution while other clades have been nearly evolutionarily static. Consistent with this, miR156–SPL interaction is extensively involved in growth and development in angiosperms (Jerome Jeyakumar *et al.* 2020). *Selaginella nipponica* has five *SPL*s in the miR156 clade; DN100333_c1_g1 and DN100480_c3_g6 are closely related to *Arabidopsis SPL13* and their expression was higher in the erect shoots, suggesting they may control the transition from vegetative to reproductive growth (Xu *et al.* 2016; He *et al.* 2018). Transcript slicing of *SPL*s by miR156 has been established in mosses (Arazi *et al.* 2005; Axtell *et al.* 2007), and this interaction plays a role in the transition of their gametophytic growth (Cho *et al.* 2012). miR156 is also found in *S*. *moellendorfii* (Axtell *et al.* 2007), and it is expressed anti-correlated with some *SPL*s discussed here; thus, it is possible that miR156-SPL drives the phase change of sporophytes evolutionarily at the first time in lycophytes. However, DN100480_c3_g6 lacks a miR156 binding site in both the coding region and 3ʹ UTR, whereas the binding motif is at most 50 bp after the stop codon in *Arabidopsis SPL3*/*4*/*5*.

*AP2*-like genes can be divided into the euAP2 and the ANT clades, and each clade contains genes from algae; thus, both clades have an origin in the common ancestor of green plants (Shigyo *et al.* 2006; Zumajo-Cardona and Pabon-Mora 2016; Kerstens *et al.* 2020; Zumajo-Cardona *et al.* 2021). In *S*. *nipponica*, DN104539_c0_g3 is closely related to the cluster of *Arabidopsis AP2*-like genes in the euAP2 clade, and it is also highly expressed in vegetative growth but not in reproductive growth, as in flowering plants (Zhu and Helliwell 2011), indicating that this gene may control the transition between growth phases. *AP2*-like genes are regulated via transcript degradation and translational repression in flowering plants, and miR172 controls these processes (Zhu and Helliwell 2011). *MIR172* originated in ancestral euphyllophytes after they had diverged from lycophytes (Zumajo-Cardona *et al.* 2021), and it can slice *AP2*-like transcripts in ferns (Axtell and Bartel 2005). Thus, *AP2*-like genes must have functioned in the absence of miR172 regulation in *S*. *nipponica*, implying a stepwise evolution of the mechanism controlling the growth phase transition of plants, which may be echoed by the unexpected lack of the miR156 binding site in one of its *SPL*s located within the miR156 clade.

## Supporting Information

The following additional information is available in the online version of this article—

**Figure S1.** The top 30 GO terms of PEGs. (A) GO terms of the prostrate shoots. (B) GO terms of the erect shoots.

**Figure S2.** The top 30 KEGG terms of PEGs. (A) KEGG terms of the prostrate shoots. (B) KEGG terms of the erect shoots.

plac018_suppl_Supplementary_Figure_S1

plac018_suppl_Supplementary_Figure_S2

## Data Availability

Sequencing data were deposited at https://www.ncbi.nlm.nih.gov/geo/query/acc.cgi?acc=GSE164112.

## Notes

**Phenome, Genome & Environment. Chief Editor**: Colleen Doherty
