# Supplementary figures and images for: Identification of genes differentially expressed between prostrate shoots and erect shoots in the lycophyte Selaginella nipponica using an RNA-seq approach
Source: AoB Plants. 2022 May 5;14(3):plac018. doi: 10.1093/aobpla/plac018 (PMC9179412; doi:10.1093/aobpla/plac018)

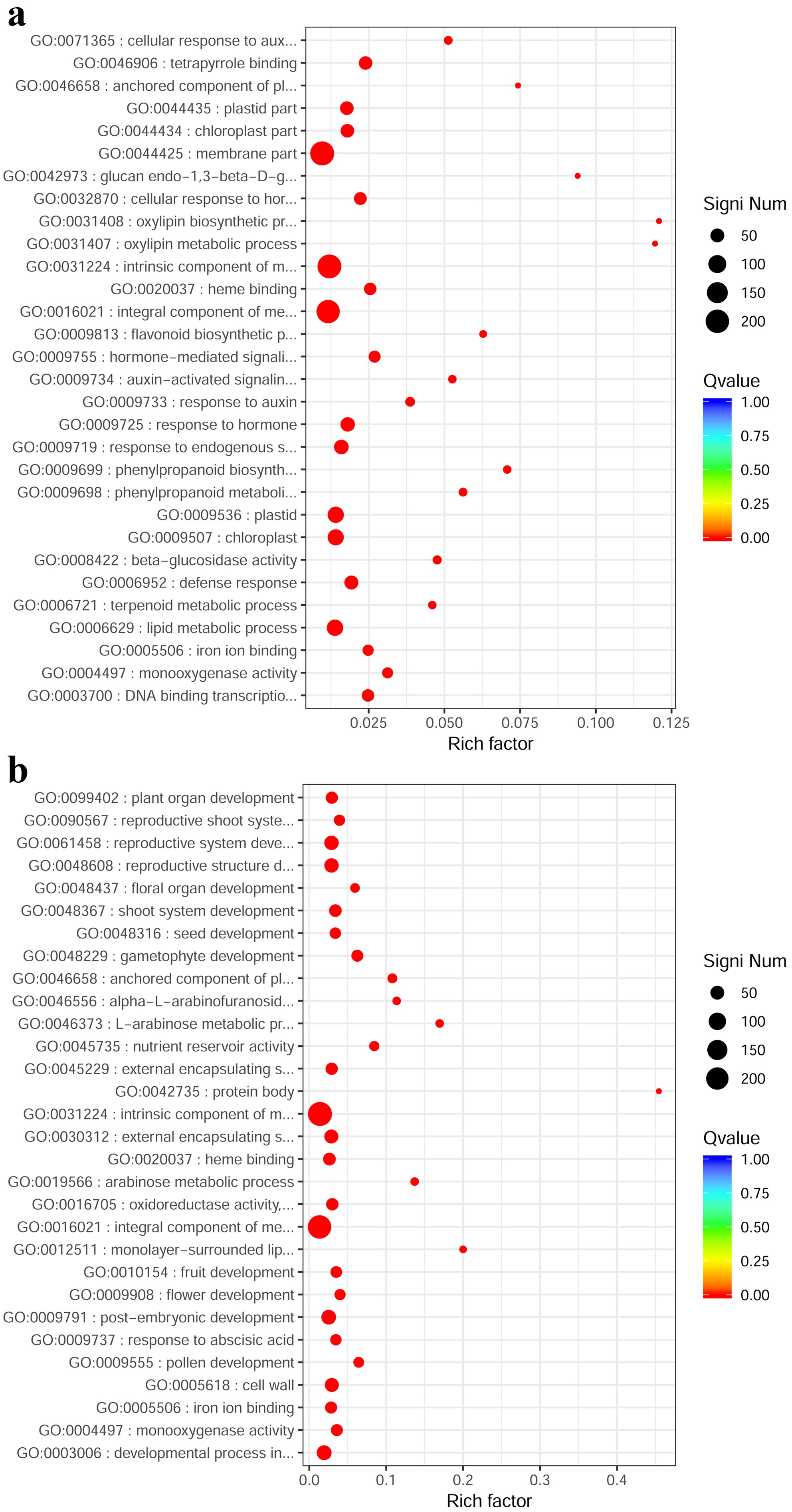

Supplement: plac018_suppl_Supplementary_Figure_S1 [file plac018_suppl_supplementary_figure_s1.jpeg]

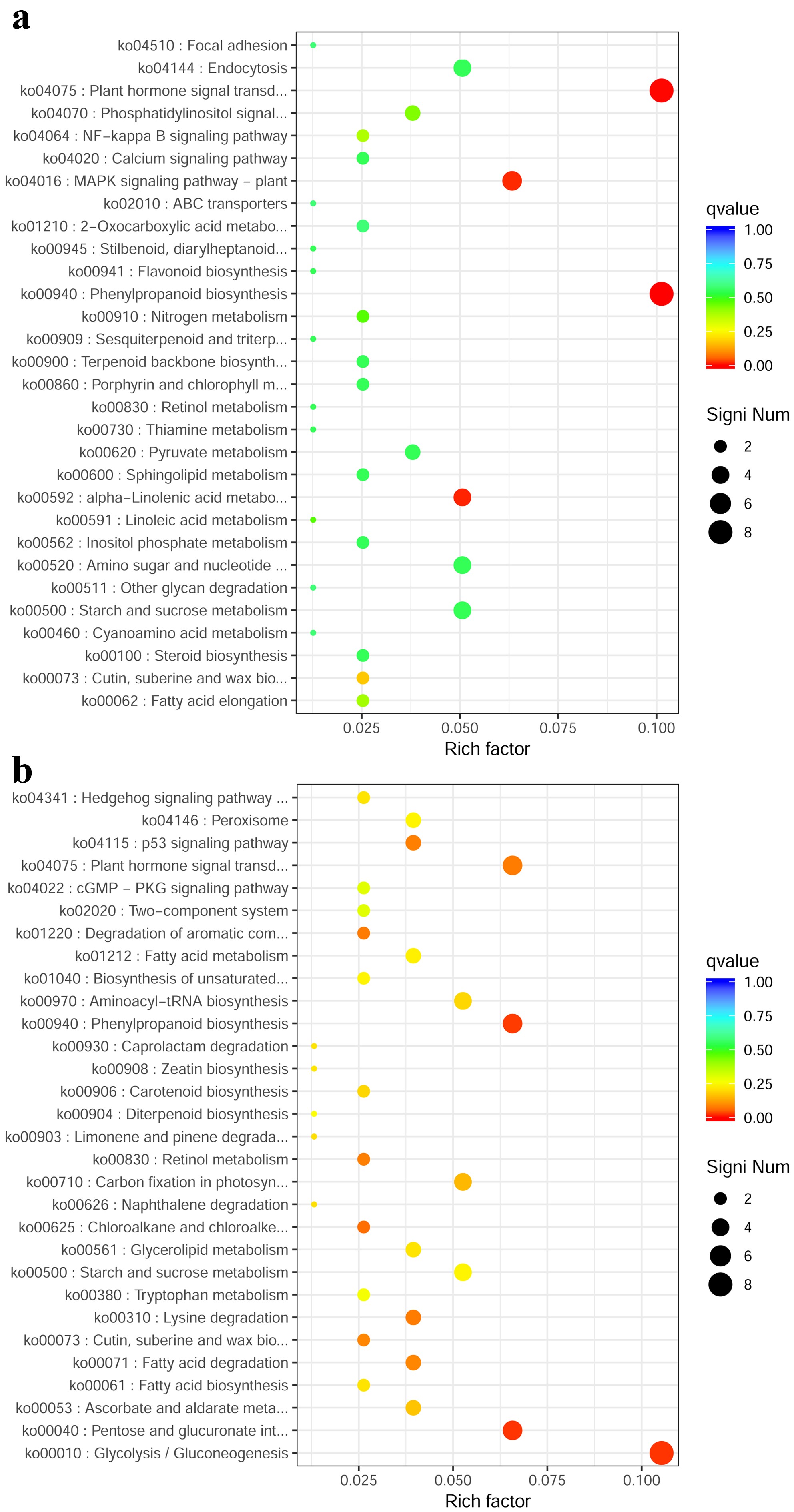

Supplement: plac018_suppl_Supplementary_Figure_S2 [file plac018_suppl_supplementary_figure_s2.jpeg]
